# 

**Published:** 1846-01

**Authors:** 


### BRITISH AND FOREIGN MEDICAL REVIEW

OR

### QUARTERLY JOURNAL

OF

### PRACTICAL MEDICINE AND SURGERY

LIBRARY OF THE

Orleans Parish Medical Society

EDITED BY

JOHN FORBES M.D. F.R.S. F.G.S.

---

VOL. XXI.

JANUARY—APRIL 1846

LONDON
JOHN CHURCHILL PRINCES STREET SOHO
MDCCCXLVI.

C. AND J. ADLARD, PRINTERS, BARTHOLOMEW CLOSE.

### BOOKS RECEIVED FOR REVIEW.

1. Spinal Affections, and the prone system of treating them. By James Coles, Surgeon. — London, 1845. 8vo, pp. 320.

2. On the Periodical Maturation and Extrusion of Ova. By Professor Bischoff. Translated by Henry Smith, Surgeon. (From the Lond. Med. Gazette.) 1845. 8vo, pp. 34.

3. The Cold-water Cure, its Use and Nature examined. By Herbert Mayo, M.D. F.R.S.— London, 1845. 8vo, pp. 84.

4. Prize Clinical Reports of Surgical Cases treated at the Queen's Hospital Birmingham. By John Moore.—London, 1845. 8vo, pp. 66.

5. The Irish Watering Places; their climate, scenery, and accommodations; including analyses of the principal Mineral Springs by Dr. R. Kane. By A. Knox, M.D.—Dublin, 1845. 8vo, pp. 332.

6. Fruits and Farinacea, the proper Food of Man. By John Smith.—Lond. 1845. 8vo.

7. On the Nature, Causes, Prevention, and Treatment of Acute Hydrocephalus. By Thomas Smith, A.M. M.D.—London, 1845. 8vo, pp. 168.

8. Introductory (Lecture) for 1844-5, On the present position of some of the most important of the modern Operations of Surgery. By J. D. Mütter, M.D.—Philadelphia, 1845. 8vo, pp. 34.

9. Some Observations on Organic Alterations of the Heart; and particularly on the beneficial Employment of Iron in the Treatment of such Cases. By S. Scott Alison, M.D.—London, 1845. 12mo, pp. 62.

10. Examination of the Views adopted by Liebig on the Nutrition of Plants. By W. Seller, M.D.— Edinburgh, 1845. 8vo, pp. 22.

11. Anecdota Sydenhamiana: Medical Notes and Observations of Thomas Sydenham, M.D., hitherto unpublished. — Oxford, 1845. 18mo, pp. 80.

12. Cosmos; a Summary of the Physical History of the Universe. By A. von Humboldt. —London, 1845. 8vo.

13. The Half-yearly Abstract of the Medical Sciences. Edited by W. H. Ranking, M.D. Vol. I. Jan.—June, 1845.—London, 1845. 8vo, pp. 390.

14. A Practical Treatise on Inflammation, Ulceration, and Induration of the Neck of the Uterus. By J. H. Bennet, M.D. &c.—London, 1845. 8vo, pp. 212.

15. The Pharmaceutical Latin Grammar; being an easy Introduction to Medical Latin. By A. J. Cooley.—London, 1845. 8vo, pp. 132.

16. A view of the Formation, Discipline, and Economy of Armies. By the late Robert Jackson, M.D. Inspector-general of Army Hospitals. The Third Edition, revised, with a Memoir of his Life and Services.—London, 1845. 8vo, pp. 426.

17. Medical and Physiological Problems. By W. Griffin, M.D. and D. Griffin, M.D.—London, 1845. 8vo, pp. 356.

18. The Nature and Treatment of Gout. By W. H. Robartson, M.D.—Lond. 1845. 8vo, pp. 372.

19. A Dictionary of Practical Medicine. By James Copland, M.D. F.R.S. &c. Part X. (*Palate —Pestilence.*) 8vo, pp. 144.

20. The History of the Middlesex Hospital during the first century of its existence. By E. Wilson, F.R.S.—London, 1845. 8vo, pp. 296.

21. Notes on a microscopical examination of Chalk and Flint. By G. A. Mantell, LL.D. F.R.S. London, 1845. 8vo, pp. 24.

22. The Ocean Flower; a Poem, preceded by an Historical and Descriptive account of the Island of Madeira. By F. M. Hughes. London, 1845. 8vo, pp. 309.

23. Urologie. Des Angusties, ou Retrecissements de l'Urêtre et de leur Traitment rationnel. Par le Dr. Leroy-d'Etiolles. Paris, 1845. 8vo, pp. 488.

24. Elements of Chemical Analysis, Qualitative and Quantitative. By E. A. Parnell. New edition, enlarged. London, 1845. 8vo, pp. 520.

25. Nuovi Elementi Fisio-Patologici di Medicina Eclettica del Dottore Niccolo Celle. Pisa, 1842. 8vo, pp. 486.

26. Metastasi riprovate dalla struttura dei Tessuti a dalla Funzioni dei Medesimi. Trattato di G. D. Bellini, M.D. Firenze, 1845. 4 fasciculi.

27. Sull' Acopuntura. Del Dr. G. B. Bellini. Fano, 1844.

28. Sui vantaggi dell' incisione laterale della bocca dell' utero nelle isterorragie per distacco di placenta, gli ultimi mesi di gravidanza. Di Dr. G. B. Bellini. 1841-45.

29. Opere di Maurizio Bufalini, Professore della Clinica Medica nelle Scuole Mediche-Chirurgiche di complimento a perfezionamento dell' Universita di Pisa in Firenze. Firenze, 1844-5. 2 vols. 8vo, pp. 378, 378.

30. Saggio Illustrativo le Tavole della Statistica Medica delle Maremme Toscane compilata. da Antonio Salvagnoli Marchetti. Firenze, 1844. Fol. pp. 86.

31. Sui Pregi e Doveri del Medico, del Professore Roberto Sava. Milano, 1845. 8vo, pp. 216.

32. Della Mortalitá e dimora Media dei Malatti nello Spedale Maggiore di Milano dal 1811 al 1 844 ed in quello dei RR. Fatebenefratelli dal 1604 al 1844 coi prospetti del calcolo complessivo sopra 784,000 infermi. Memoria del Medico-Statista Dottore Giuseppe Ferrario. Milano, 1845. 8vo, pp. 16.

33. The Modern Treatment of Syphilitic Diseases, both Primary and Secondary. By Langston Parker, Surgeon to the Queen's Hospital, Birmingham. Second Edition. London, 1845. 8vo, pp. 228.

34. First Steps to Anatomy. By J. L. Drummond, M.D. London, 1845. 8vo, pp. 201.

35. Homœopathic Domestic Medicine. By J. Laurie, M.D. Third Edition. London, 1846. 8vo, pp. 576.

36. On Scarlatina, and its successful treatment by the Acidum Aceticum dilutum of the Pharmacopœia. By I. B. Brown. London, 1846. 8vo, pp. 66.

37. Lectures on Natural and Difficult Parturition. By E. W. Murphy, A.M., M.D., Professor of Midwifery, University College. London, 1845. 8vo, pp. 263.

